# Development and validation of a predictive model for oral mucosal pressure injury risk in ICU patients with endotracheal intubation

**DOI:** 10.3389/fmed.2025.1695085

**Published:** 2025-12-18

**Authors:** Limei Cai, Yijing Li, Meng Zheng, Yonggang Liu, Guo Ma, Qinfang Zhang, Xiaoxi Li, Na Li

**Affiliations:** 1 Department of Intensive Care Medicine, The First Affiliated Hospital of Kunming Medical University, Kunming, Yunnan, China; 2 Nursing Department rotation, Kunming Yan’an Hospital, Kunming, Yunnan, China; 3 Department of Dermatology, The First Affiliated Hospital of Kunming Medical University, Kunming, Yunnan, China

**Keywords:** intensive care unit, tracheal intubation, oral mucosal pressure injury, predictive model, logistic regression

## Abstract

**Objective:**

To identify risk factors for oral mucosal pressure injury (OMPI) in intensive care unit (ICU) patients undergoing orotracheal intubation and to develop and validate a risk prediction nomogram based on logistic regression analysis.

**Methods:**

Relevant risk factors for OMPI were identified through a combination of literature review and expert interviews. A total of 426 intubated ICU patients admitted to a tertiary hospital in Yunnan Province between May and December 2024 were included in the model group. Variables with *P* < 0.10 in univariate analysis were further entered into multivariate logistic regression to identify independent risk factors for OMPI and construct a predictive nomogram. Missing data were addressed using multiple imputation, and potential confounders such as age, BMI, and disease severity were adjusted for in the multivariable analysis. Model performance was evaluated by the area under the ROC curve (AUC), calibration plots, and decision curve analysis (DCA), and internally validated using bootstrap resampling. An external validation cohort of 152 patients from January to March 2025 was used to assess the model’s predictive performance. All analyses were performed using SPSS version 27.0 and R version 4.3.2, with a two-tailed *P* < 0.05 considered statistically significant.

**Results:**

Duration of intubation, use of dental pads, Richmond Agitation-Sedation Scale (RASS) score, Brachycephalic Obstructive Airway Syndrome (BOAS) score, and platelet count were identified as independent risk factors for OMPI (*P* < 0.01). The model showed good discriminative ability with an AUC of 0.888 (95% CI: 0.849–0.926). The calibration curve demonstrated strong agreement between predicted and observed outcomes, and the Hosmer–Lemeshow test indicated good calibration (χ^2^ = 3.95, *P* = 0.861). DCA showed net clinical benefit within a 3–100% risk threshold. External validation yielded an AUC of 0.854, sensitivity of 86.5%, specificity of 73.0%, and overall predictive accuracy of 83.7%.

**Conclusion:**

The validated nomogram demonstrated good discrimination, calibration, and clinical utility, offering a reliable tool for early identification of high-risk ICU patients and for guiding personalized interventions. Nevertheless, as this was a single-center study, further multicenter validation is needed to confirm its generalizability.

## 1 Introduction

With advances in critical care medicine and airway management, orotracheal intubation has become one of the essential interventions for the resuscitation and treatment of critically ill patients in intensive care units (ICUs). It effectively maintains airway patency and supports survival in critically ill patients (1). Oral mucosal pressure injury (OMPI) is a type of medical device-related pressure injury (MDRPI) (2). Due to prolonged oral opening and reduced mucosal defense, patients undergoing orotracheal intubation are susceptible to OMPI, which is often induced by sustained pressure and friction from medical devices such as dental pads, endotracheal tubes, and tube fixators (3). ICU patients are particularly vulnerable due to factors such as altered consciousness, immobility, malnutrition, and poor perfusion (4). Studies have reported the incidence of OMPI following intubation to range from 2.59 to 48.80% (5, 6). OMPI increases pain, infection risk, and nursing workload, underscoring the need for early identification and timely intervention (7). Currently, there is no standardized tool for OMPI risk assessment; most studies focus on isolated factors, while existing scales such as Braden and Norton overlook oral-specific variables like mucosal fragility, tube fixation, and sedation level. In clinical practice, integrating a predictive model into ICU nursing workflows could support early identification of high-risk patients, guide targeted oral care interventions, and optimize resource allocation. The use of a nomogram provides an intuitive visualization of individual risk, enabling bedside nurses and clinicians to make rapid, evidence-based decisions in the complex ICU setting. Therefore, this study aimed to develop and validate a nomogram integrating multiple clinical predictors to enable early risk identification in intubated ICU patients.

This study aims to identify potential risk factors for OMPI through literature review and expert consultation, and to construct a predictive model using retrospective clinical data and logistic regression analysis. The resulting model is visualized as a nomogram. Model performance is evaluated through internal and external validation to assess its discriminative power, calibration, and clinical utility, with the ultimate goal of offering ICU clinicians a precise risk alert tool to optimize patient care.

## 2 Materials and methods

### 2.1 Participants

This was a prospective observational cohort study that adopted a convenience sampling method to recruit ICU patients who underwent orotracheal intubation between May and December 2024.

Inclusion criteria were as follows:

(1)    patients receiving mechanical ventilation via orotracheal intubation;
(2)    aged ≥ 18 years;
(3)    intubation duration > 24 h;
(4)    informed consent provided by the patients or their legal representatives, with voluntary participation in the study.

Exclusion criteria included:

(1)    patients with pre-existing oral mucosal or skin damage before intubation;
(2)    patients with mucosal defects resulting from trauma or oral and maxillofacial surgery;
(3)    patients with incomplete clinical data.

Based on prior literature review and expert panel discussions, and in alignment with clinical practice, a total of 31 risk factors were identified for inclusion. The required sample size was calculated using the formula for observational studies based on population rate estimation (8): *n* = Z^2^_α/2_P (1-P)/δ^2^, in which Zα/2 was the percentile value under the standard normal distribution corresponding to cumulative probability α/2 at α = 0.05, with Z_α/2_ equal to 1.96; δ was set at 5%, and *P* (estimated incidence) was drawn from existing literature, which reported OMPI incidence ranging from 2.59 to 48.80% in intubated ICU patients (5, 6). Considering a 10% invalid data rate, the calculated sample size ranged from 48 to 426 cases. Ultimately, 426 patients were included in the modeling cohort. An external validation cohort comprised 152 intubated patients admitted to the same ICU from January to March 2025.

The study was approved by the institutional ethics committee (Approval No. 2024 Ethics Review L No. 265).

### 2.2 Methods

#### 2.2.1 Research tools

A systematic literature search was conducted in domestic and international databases to identify studies examining risk factors for oral mucosal injury in intubated patients. Articles were screened and critically appraised for quality. Due to the heterogeneity in definitions and the wide range of risk factors identified across studies, meta-analysis was deemed unsuitable, and a qualitative synthesis approach was adopted instead.

Using NVivo 14 qualitative analysis software, thematic coding and clustering visualization of OMPI-related factors were performed (9). After summarization and categorization, five primary thematic nodes were established: patient-related factors, device-related factors, treatment-related factors, disease-related factors, and physiological/biochemical indicators. An initial draft of the risk factor survey form was developed based on these findings. The form was finalized through expert panel discussions.

The severity of OMPI was assessed using the Reaper Oral Mucosa Pressure Injury Scale (ROMPIS), developed by Reaper et al. (10).

#### 2.2.2 Data collection

Trained members of the research team collected data within 24 h of ICU admission. Data on endotracheal tube type and material, use of bite blocks, fixation method, and presence of mucosal injury were obtained through direct observation. Demographic, clinical, and laboratory data were extracted from electronic medical records. Oral mucosal assessments were performed by researchers at 06:00, 14:00, and 22:00 during routine oral care. OMPI staging was performed using the ROMPIS. ICU nurses who received standardized training assessed oral mucosa after fixation removal and oral care, following a uniform ROMPIS-based protocol. Two assessors worked in pairs and cross-checked results to ensure reliability. In cases of diagnostic uncertainty regarding injury staging, group consensus or consultation with relevant specialists was sought.

#### 2.2.3 Statistical analysis

Data were independently entered by two individuals and analyzed using SPSS version 27.0. For univariate analysis of risk factors associated with OMPI in intubated patients, normally distributed continuous variables were expressed as mean ± standard deviation (x ± s), and between-group comparisons were conducted using the *t*-test. Categorical variables were presented as counts and percentages, with comparisons performed using the χ^2^ test. Non-normally distributed continuous variables were expressed as median and interquartile range [M (Q1, Q3)], and compared using non-parametric tests. Prior to analysis, continuous variables were tested for normality. Normally distributed data were expressed as mean ± standard deviation and compared using the *t*-test, while non-normally distributed data were expressed as median (Q1, Q3) and compared using the Mann–Whitney U test. Categorical variables were compared using the χ^2^-test. Results of logistic regression were expressed as odds ratios (ORs) with 95% confidence intervals (CIs). Residual analysis was not performed and is acknowledged as a limitation. Missing data were handled using multiple imputation to reduce potential bias. To control for confounding, clinically relevant variables such as age, disease severity, BMI, and medical history were predetermined as potential confounders and were forced into the multivariable model as covariates.

## 3 Results

### 3.1 General characteristics of the study population

In the modeling cohort, a total of 426 patients underwent orotracheal intubation, among whom 172 developed OMPI, yielding an incidence rate of 40.37%. The majority of these injuries were classified as stage II (130 cases, 66.67%), followed by stage I (25 cases, 14.5%) and stage III (17 cases, 9.9%).

Regarding demographic characteristics, most patients were middle-aged or elderly (aged 50–70 years), accounting for 184 cases (43.20%). There were 130 female patients (30.5%) and 296 male patients (69.5%). The majority of patients had a normal body mass index (BMI), with 256 cases (60.1%).

In terms of disease-related information, gastrointestinal disorders were the most common primary diagnosis, observed in 156 cases (36.6%). For intubation-related characteristics, a 7.5# endotracheal tube was most frequently used (288 cases, 67.6%), and the majority of tubes were wire-reinforced (235 cases, 55.2%). The median intubation duration was 8 days, with an interquartile range (IQR) of 3–13 days. The most common fixation method was a combination of adhesive tape and tie (245 cases, 57.5%). Most patients (270 cases, 63.4%) used bite blocks.

In the validation cohort, there were 152 patients in total, with 63 cases (41.45%) developing OMPI. Most patients were between 50 and 70 years old (63 cases, 37.50%). The cohort included 54 female patients (35.53%) and 98 male patients (64.47%).

### 3.2 Univariate analysis of risk factors for OMPI in ICU patients undergoing endotracheal intubation

According to the results of univariate analysis, the following factors showed statistically significant differences between patients with and without OMPI (all *P* < 0.05) (see Table 1): length of ICU stay, duration of endotracheal intubation, primary diagnosis, use of bite blocks, APACHE II score, Richmond Agitation-Sedation Scale (RASS) score, Brachycephalic Obstructive Airway Syndrome (BOAS) score, administration of anticoagulant therapy, and platelet count.

**TABLE 1:** Univariate analysis of risk factors for oral mucosal pressure injury in intubated patients in the modeling cohort (*n* = 426).

Variable	OMPI group (*n* = 172)	Non-OMPI group (*n* = 254)	t/Z/χ^2^-value/	*P*
Age (years)			2.518	0.472
18–30	14 (8.1%)	26 (10.2%)		
30–50	44 (25.6%)	50 (19.7%)		
50–70	70 (40.7%)	114 (44.9%)		
≥ 70	44 (25.6%)	64 (25.2%)		
Body mass index (BMI, kg/m^2^)			1.621	0.445
< 18.5	22 (12.8%)	24 (9.4%)		
18.5–23.9	104 (60.5%)	152 (59.8%)		
≥ 23.9	46 (26.7%)	78 (30.7%)		
Gender			0.285	0.594
Male	122 (70.9%)	174 (68.5%)		
Female	50 (29.1%)	80 (31.5%)		
Primary diagnosis			45.770	< 0.001
Cardiovascular system	10 (5.8%)	14 (5.5%)		
Respiratory system	36 (20.9%)	18 (7.1%)		
Neurological system	60 (34.9%)	50 (19.7%)		
Gastrointestinal system	36 (20.9%)	120 (47.2%)		
Hematologic system	2 (1.2%)	6 (2.4%)		
Musculoskeletal system	6 (3.5%)	14 (5.5%)		
Other	22 (12.8%)	32 (12.6%)		
Tube size			3.807	0.149
7.0#	42 (24.4%)	72 (28.3%)		
7.5#	116 (67.4%)	172 (67.7%)		
8.0#	14 (8.1%)	10 (3.9%)		
Tube material			0.594	0.441
Standard PVC	81 (47.1%)	110 (43.3%)		
Wire-reinforced	91 (52.9%)	144 (56.7%)		
ICU length of stay (days)	18 (12,24)	8 (4,16)	–8.825	< 0.001
Intubation duration (days)	12(9,14)	5 (3,10)	–9.707	< 0.001
Fixation method			0.325	0.850
Tape only	69 (40.1%)	104 (40.9%)		
Tape + tie	99 (57.6%)	146 (57.5%)		
Tie only	4 (2.3%)	4 (1.6%)		
Bite block use			21.145	< 0.001
No	44 (25.6%)	112 (44.1%)		
Yes	128 (74.4%)	142 (55.9%)		
Subglottic suctioning			0.475	0.491
No	166 (96.5%)	248 (97.6%)		
Yes	6 (3.5%)	6 (2.4%)		
Apache II score	25 (22,27)	24(20.75,26.00)	–2.170	0.03
RASS score			44.410	< 0.001
Agitation (+ 2– + 4)	22 (12.8%)	2 (0.8%)		
Light sedation (−2– + 1)	42 (24.4%)	120 (47.2%)		
Moderate sedation (−3–4)	65 (37.8%)	100 (39.4%)		
Deep sedation (−5)	43 (25.0%)	32 (12.6%)		
BOAS score			39.234	< 0.001
5 (Normal)	6 (3.5%)	36 (14.2%)		
6–10 (Mild impairment)	80 (46.5%)	176 (69.3%)		
11–15 (Moderate impairment)	78 (45.3%)	42 (16.5%)		
16–20 (Severe impairment)	8 (4.7%)	0 (0.0%)		
Braden score	12 (11,12.75)	12 (11,13)	−1.2230	0.221
Pressure ulcer stage			5.658	0.341
No ulcer	128 (74.4%)	203 (79.9%)		
Stage I	12 (11.6%)	28 (11.0%)		
Stage II	20 (7.0%)	16 (6.3%)		
Stage III	6 (3.5%)	2 (0.8%)		
Unstageable	2 (1.2%)	1 (0.4%)		
Suspected deep tissue injury	4 (2.3%)	4 (1.6%)		
Edema			0.981	0.322
No	146 (84.9%)	224 (88.2%)		
Yes	26 (15.1%)	30 (11.8%)		
History of cardiovascular disease			1.664	0.197
No	116 (67.4%)	186 (73.2.9%)		
Yes	56 (32.6%)	68 (26.8%)		
History of diabetes			0.690	0.406
No	144 (83.7%)	220 (86.6%)		
Yes	28 (16.3%)	34 (13.4%)		
Vasopressor use			0.024	0.876
No	84 (48.8%)	126 (49.6%)		
Yes	88 (51.2.9%)	128 (50.4%)		
Corticosteroid use			2.379	0.123
No	145 (84.3%)	227 (89.4%)		
Yes	27 (15.7%)	27 (10.6%)		
Anticoagulant use			5.584	0.018
No	146 (84.9%)	234 (92.1%)		
Yes	26 (15.1%)	20 (7.9%)		
Analgesic use			1.638	0.201
No	6(3.5%)	4 (1.6%)		
Yes	166 (96.5%)	250 (98.4%)		
Sedative use			0.004	0.953
No	56 (32.6%)	82 (32.3%)		
Yes	116 (67.4%)	172 (67.7%)		
Prone ventilation			2.538	0.111
No	165 (95.9%)	250 (98.4%)		
Yes	7 (4.1%)	4 (1.6%)		
ECMO treatment			2.851	0.091
No	167 (97.1%)	252 (99.2%)		
Yes	5 (2.9%)	2 (0.8%)		
Serum albumin (g/L)	31.50 ± 6.31	31.49 ± 6.11	0.014	0.989
Hemoglobin (g/L)	109.31 ± 28.96	109.03 ± 28.96	0.103	0.989
Hematocrit (%)	32.93 ± 8.63	33.07 ± 8.05	−0.171	0.864
Platelet count (10^∧^9/L)			29.845	< 0.001
> 100	113 (65.7%)	215 (84.6%)		
50–100	29 (16.9%)	31 (12.2%)		
≤ 50	30 (17.4%)	8 (3.1%)		
Oxygenation index (mmHg)			4.342	0.114
< 200	187 (43.9%)	108 (42.5%)		
200–300	162 (38.0%)	92 (36.2%)		
≥ 300	77 (18.1%)	54 (21.3%)		

RASS, Richmond Agitation-Sedation Scale; PVC, polyvinyl chloride; BOAS, Brachycephalic Obstructive Airway Syndrome.

### 3.3 Multivariate analysis of oral mucosal pressure injury in ICU patients undergoing tracheal intubation

The dependent variable Y indicated the occurrence of oral mucosal injury, coded as 0 for absence and 1 for presence. Variables with a *P* < 0.10 in univariate analysis—including ICU length of stay, duration of intubation, diagnosis, use of a mouthguard, APACHE II score, RASS score, BOAS score, ECMO therapy, anticoagulant use, and platelet count—were included as independent variables (X). Before model construction, multicollinearity was assessed using tolerance and variance inflation factor (VIF), with tolerance > 0.1 and VIF < 10 as acceptable thresholds. Tolerance ranged from 0.381 to 0.951, and VIF ranged from 1.038 to 2.627, indicating no severe multicollinearity among variables. Residual analysis was not conducted, which is acknowledged as a limitation. Subsequently, statistically significant variables from the univariate analysis were entered into a binary logistic regression model using the backward stepwise (Wald) method. The results demonstrated that intubation duration [OR = 1.254, 95% CI (1.184–1.330)], mouthguard use [OR = 3.531, 95% CI (2.029–6.143)], RASS score [OR = 0.605, 95% CI (0.436–0.840)], BOAS score [OR = 3.255, 95% CI (2.209–4.794)], and platelet count [OR = 2.269, 95% CI (1.480–3.478)] were independent risk factors associated with oral mucosal injuries (all *P* < 0.05) (see Table 2).

**TABLE 2:** Multivariate logistic regression analysis of risk factors for oral mucosal pressure injury in ICU patients undergoing tracheal intubation.

Variable	*β*	*SE*	Wald	*P*	*OR*	95%CI	
						Lower limit	Upper limit
Duration of intubation (days)	0.227	0.030	58.326	<0.001	1.254	1.184	1.330
Use of mouthguard	1.262	0.283	19.933	<0.001	3.531	2.029	6.143
RASS score	−0.502	0.167	9.024	0.003	0.605	0.436	0.840
BOAS score	1.180	0.198	35.662	<0.001	3.255	2.209	4.794
Platelet count	0.819	0.218	14.128	<0.001	2.269	1.480	3.478
Constant	−5.547	0.689	64.888	<0.001	0.004		

β, regression coefficient; SE, Standard Error; Wald, Wald chi-square Statistic; OR, Odds Ratio; CI, Confidence Interval; RASS, Richmond Agitation-Sedation Scale; BOAS, Brachycephalic Obstructive Airway Syndrome.

### 3.4 Construction of a nomogram model for predicting the risk of oral mucosal pressure injury in ICU patients undergoing tracheal intubation

**FIGURE 1:**
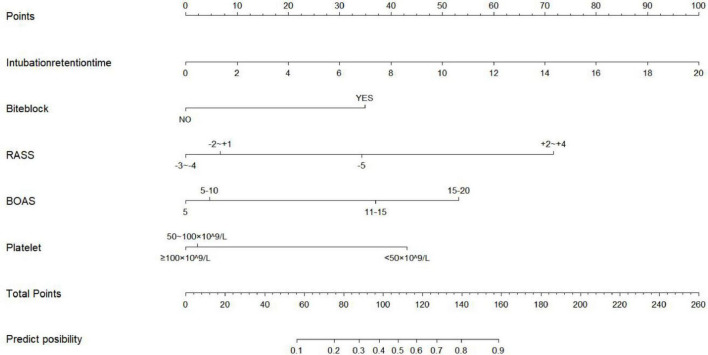
Nomogram for predicting the risk of oral mucosal pressure injury (OMPI) in ICU patients undergoing tracheal intubation (*n* = 426). The model was built using multivariable logistic regression with five predictors: intubation retention time, bite block use, RASS score, BOAS score, and platelet count. Each predictor is assigned a point value on the upper scale, and the total points correspond to the predicted probability of OMPI at the bottom. RASS, Richmond Agitation–Sedation Scale; BOAS, Beck Oral Assessment Scale.

Based on the results of the logistic regression analysis, a nomogram for predicting the risk of OMPI in intubated ICU patients was developed using the rms package in R software (Figure 1).

### 3.5 Evaluation and validation of the prediction model for OMPI in ICU patients undergoing orotracheal intubation

#### 3.5.1 Model evaluation and internal validation

**FIGURE 2:**
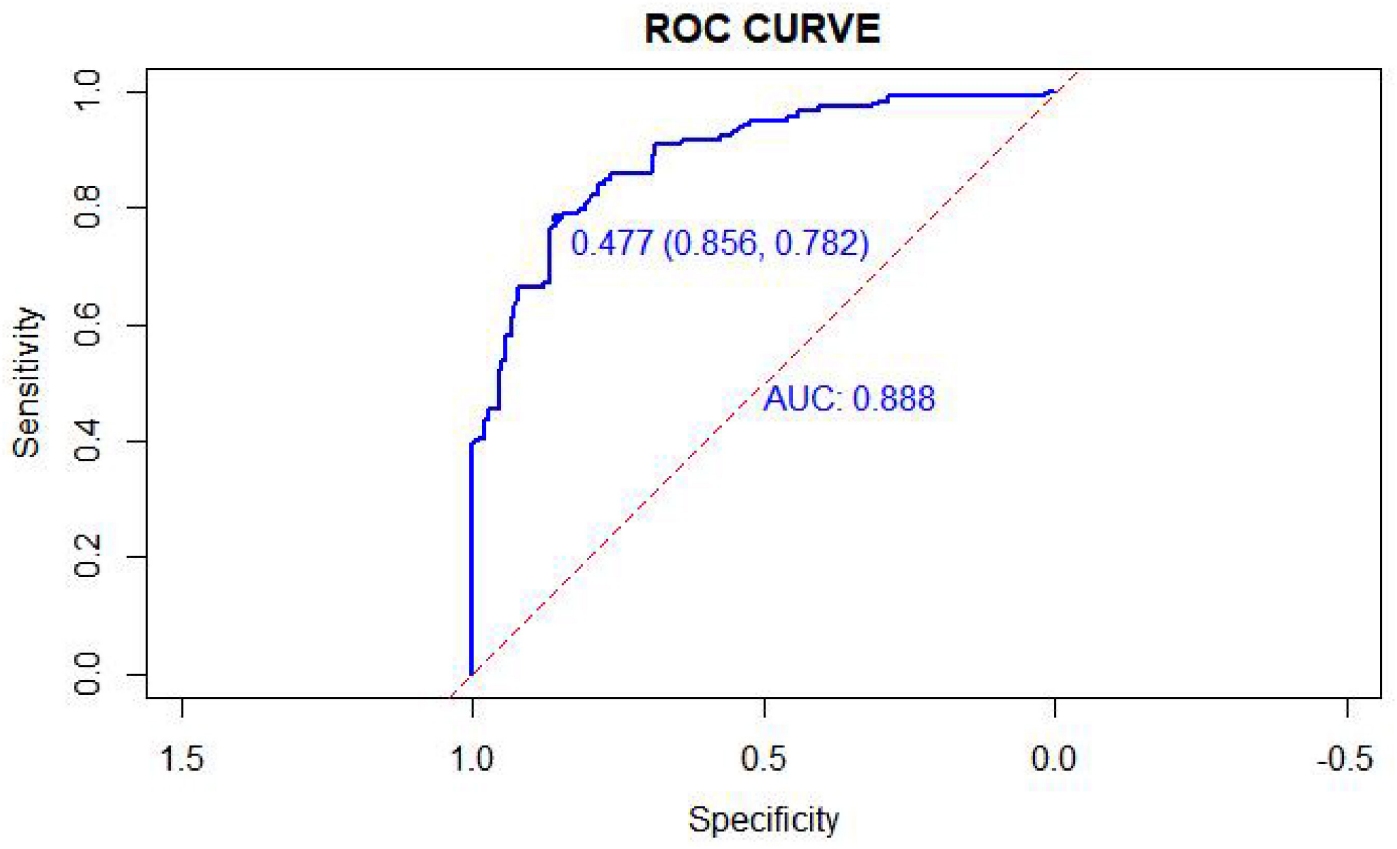
Receiver operating characteristic (ROC) curve of the prediction model for OMPI in the training cohort (*n* = 426). The model achieved an AUC = 0.888 (95% CI 0.849–0.926) with an optimal cutoff value = 0.477 (sensitivity = 78.2%, specificity = 85.6%). AUCs were estimated using the non-parametric DeLong method. ROC, receiver operating characteristic; AUC, area under the curve.

The nomogram-based risk prediction model for OMPI in ICU patients undergoing orotracheal intubation demonstrated good discriminative ability in the training cohort, with an area under the receiver operating characteristic (ROC) curve (AUC) of 0.888 (95% CI: 0.849–0.926) (Figure 2). The optimal cutoff risk value was 0.477, yielding a specificity of 85.6%, sensitivity of 78.2%, and a Youden index of 0.638.

**FIGURE 3:**
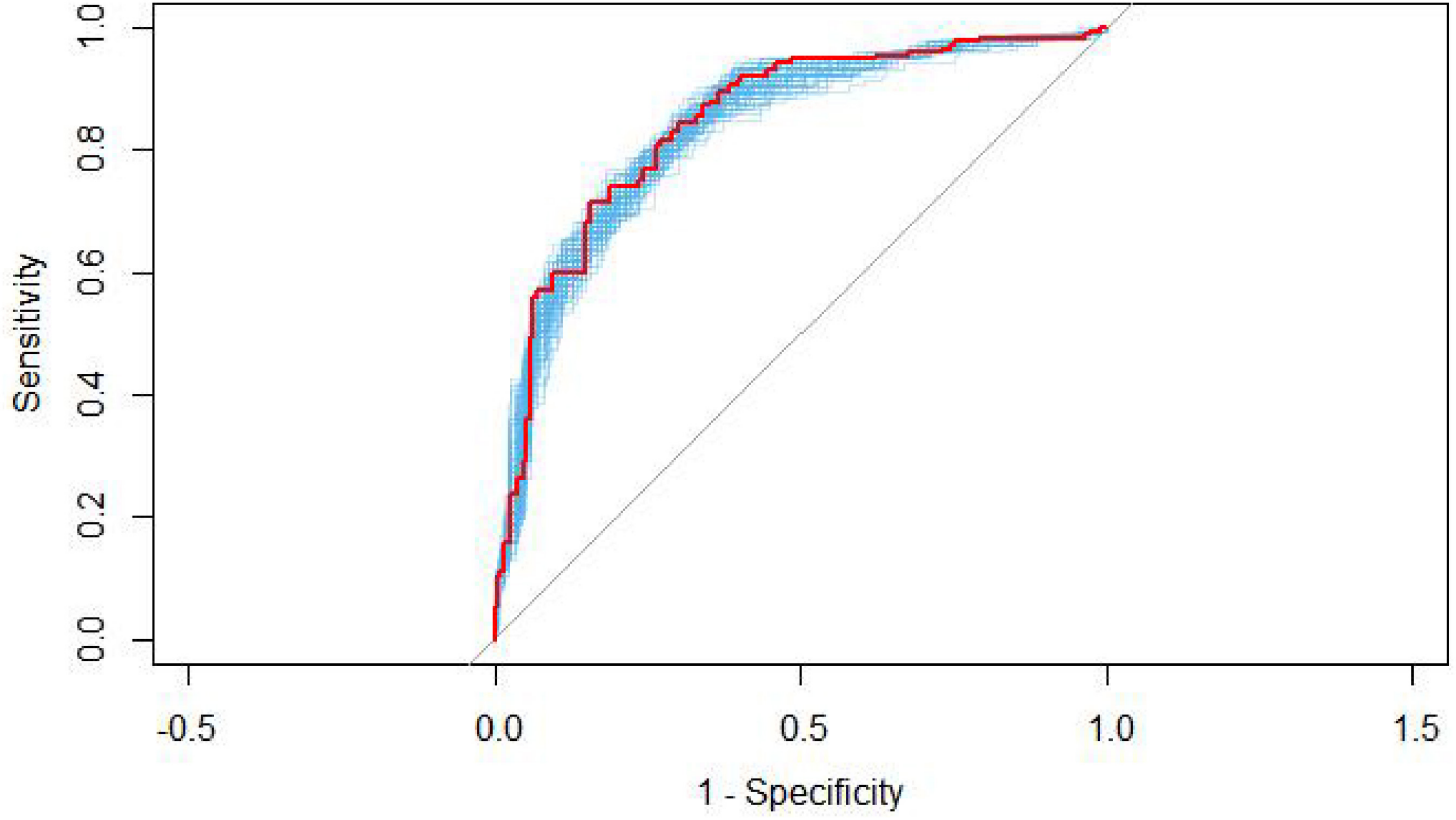
Internal validation of the predictive model using 1,000 bootstrap resampling iterations based on the training cohort (*n* = 426). The internally validated AUC was 0.867 (95% CI 0.832–0.901), indicating consistent discrimination after resampling.

**FIGURE 4:**
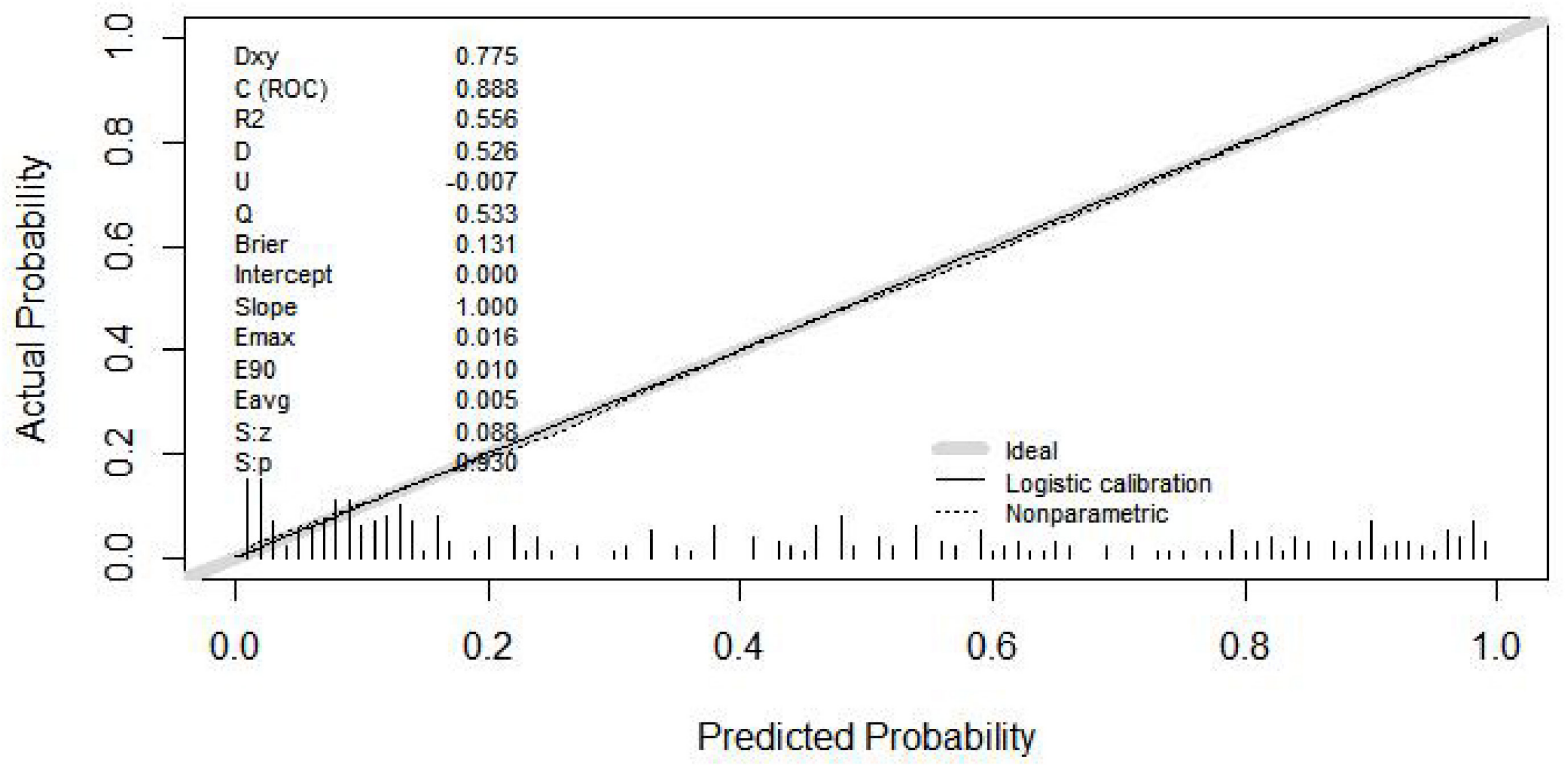
Calibration curve of the nomogram in the training cohort (*n* = 426) based on 1,000 bootstrap resamples. The solid line represents the model’s predicted probabilities versus the observed outcomes, while the dashed line denotes the ideal calibration. The model demonstrated good calibration (Hosmer–Lemeshow χ^2^ = 3.95, *P* = 0.861; Brier score = 0.131).

Internal validation was conducted using 1,000 bootstrap resampling iterations. The resulting AUC from the internally validated ROC curve (Figure 3) was 0.867 (95% CI: 0.832–0.901), indicating consistent performance of the model. The Hosmer–Lemeshow goodness-of-fit test yielded a chi-square value of 3.950 with a *P*-value of 0.861 (*P* > 0.05), suggesting good calibration in the training set. The calibration curve generated using 1,000 bootstrap samples (Figure 4) showed good agreement between the predicted and observed probabilities, indicating adequate model calibration.

#### 3.5.2 External validation

**FIGURE 5:**
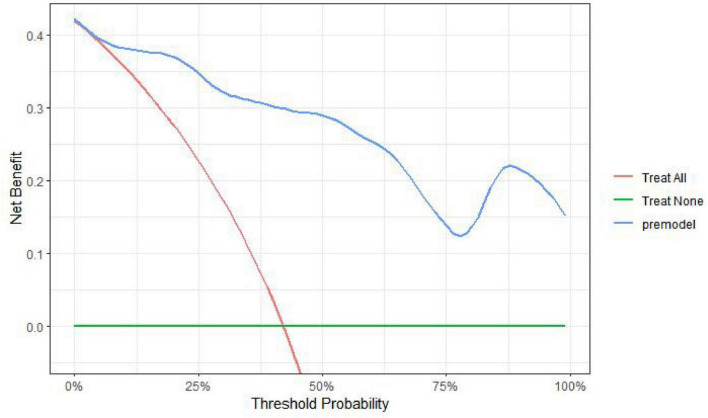
Decision curve analysis (DCA) of the predictive model in the external validation cohort (*n* = 152). The blue line (model) remains above both the “treat-all” (red) and “treat-none” (green) strategies across threshold probabilities of 3–100%, indicating a favorable net clinical benefit for predicting OMPI risk.

External validation of the model was performed on a validation cohort comprising 152 ICU patients who underwent orotracheal intubation. The AUC of the ROC curve for this cohort was 0.854 (95% CI: 0.795–0.914), with a sensitivity of 0.865, specificity of 0.730, and an overall accuracy of 83.7%. The clinical utility of the prediction model was evaluated using DCA (Figure 5). The DCA curve remained above both the “treat-all” and “treat-none” extremes across a wide range of threshold probabilities (Pt: 0.03–1), indicating that the model provides a favorable net clinical benefit and has high applicability for predicting OMPI in ICU patients undergoing orotracheal intubation.

## 4 Discussion

### 4.1 Incidence of oral mucosal pressure injury in patients undergoing tracheal intubation

The findings of this study revealed that the incidence of OMPI among ICU patients in the training cohort was 40.37%, with most injuries classified as Stage II. This result is consistent with that reported by Wang (11) (44.69%) and Coyer et al. (12) (41.6%). However, the incidence observed in this study is higher than those reported by Peking Union Medical College (13) (25.9%) and Kim et al. (14) (36.3%). The variation may be attributed to differences in study populations and sample sizes. In this study, patients were admitted to a general ICU with diverse diagnoses, including severe traumatic brain injury, acute severe pancreatitis, and severe pulmonary infections, which are characterized by critical conditions, prolonged courses, and severe infections (15). These factors may contribute to extended intubation durations and increased OMPI risk. Inconsistencies in assessment tools and grading criteria may also account for the differences. This study used the Reaer et al. scoring scale, whereas Wang (16) adopted the WHO grading standard. A unified evaluation tool is currently lacking. Furthermore, intubation duration and fixation methods may influence injury risk. The median intubation duration in this study was 8 days (IQR: 3–13), which is relatively long. Fixation was based on the patient’s level of consciousness and cooperation. For agitated patients, the tube was secured using ties in combination with a standard mouthguard, which increases mucosal pressure and friction, thereby elevating the risk of injury. In summary, multiple factors contribute to OMPI in intubated patients. Differences in populations, methodologies, and evaluation criteria result in varying incidence rates across studies. Additionally, factors such as oral secretion accumulation or fixation pressure may contribute to the occurrence of OMPI in critically ill patients, although these aspects were not directly evaluated in this study and should be examined in future research. The type of primary disease also influences OMPI susceptibility. Patients with severe neurological or gastrointestinal disorders often experience malnutrition, prolonged immobilization, and circulatory compromise, which collectively impair mucosal resilience and delay healing under device pressure.

### 4.2 Independent risk factors for oral mucosal pressure injury in patients with tracheal intubation

#### 4.2.1 Duration of tracheal intubation

The longer the tracheal tube remains in place, the greater the risk of developing OMPI. One study (17) reported that each additional day of intubation doubles the risk of oral mucosal injury. Another study (18) also identified prolonged intubation (OR = 2.321) as a significant risk factor, consistent with the findings of the present study. Research has shown (19) that mucosal ischemia becomes irreversible after as little as 2 h of sustained pressure. Continuous pressure from the endotracheal tube and its securing device can lead to local ischemia and necrosis of the oral mucosa, manifested as erythema, erosion, ulceration, or bleeding. Therefore, it is essential to optimize fixation methods, select appropriate securing devices, and avoid overly tight or loose placement. Regular repositioning of the tube should be performed to reduce continuous pressure on the same site. For patients whose clinical condition permits, early weaning and extubation should be encouraged to minimize the duration of mucosal compression. Generally, oral endotracheal tubes should not remain in place for more than 2 weeks. For patients requiring long-term airway support, tracheostomy should be considered to reduce the risk of complications (20). Additionally, the use of prophylactic dressings or cushioning materials may help protect the oral mucosa from friction and pressure in patients who require prolonged intubation.

#### 4.2.2 Use of mouthguards

Mouthguards are commonly used to secure endotracheal tubes, prevent displacement or structural damage, and protect the tube from being bitten (21). However, their rigidity and shape may exert direct pressure or cause abrasion on the oral mucosa, particularly when used over extended periods, increasing the risk of mucosal breakdown (16) . In this study, the use of mouthguards was identified as an independent risk factor for OMPI, with a 3.531-fold higher risk compared to those not using them. This is consistent with the findings of Kim et al. (14), but differs from the results of Gu (22), whose patients used silicone mouthguards, which are softer and exert less mechanical friction. In contrast, this study involved conventional mouthguards made of harder materials with sharp edges, which increase shear and frictional forces, especially in agitated patients who frequently bite the tube. Recent studies have proposed improvements in mouthguard design (23), focusing on modifications to shape and material. Moreover, for conscious or edentulous elderly patients, it is recommended to secure the tube without a mouthguard whenever feasible (24). These strategies aim to reduce mucosal injury while ensuring patient safety.

#### 4.2.3 RASS score

The Richmond Agitation–Sedation Scale (RASS) score was identified as an independent risk factor for OMPI in this study. Patients with agitation (RASS + 2– + 4) were at the highest risk, followed by those with deep sedation (−5), light sedation (−2– + 1), and moderate sedation (−3–−4). This is consistent with the findings of Kim et al. (14), which indicated that patients not receiving sedatives were more likely to experience mucosal injuries due to spontaneous movements such as swallowing, biting, or attempting to expel the tube or mouthguard, thus increasing pressure and friction on the mucosa (25). Choi et al. (26) similarly found that sedative use reduces motor activity, muscle tone, and sensory perception, limiting patients’ ability to express discomfort due to excessive pressure, thereby contributing to OMPI. Therefore, for mechanically ventilated patients not scheduled for immediate extubation, appropriate use of sedatives and analgesics is recommended to reduce agitation, pain, and ventilator–patient asynchrony. Patients with low consciousness or deep sedation lack protective reflexes and pain perception, allowing prolonged localized compression by the endotracheal tube. This physiological reduction in self-adjustment increases mucosal ischemia risk and explains the observed association between lower RASS scores and OMPI incidence. The findings suggest that maintaining an appropriate sedation depth through regular assessment and timely dose adjustment may help minimize the risk of mucosal injury in mechanically ventilated patients (27).

#### 4.2.4 BOAS score

This study found that oral health status, as measured by the BOAS score, was significantly associated with the occurrence of OMPI. A higher BOAS score correlated with a greater risk of OMPI [OR = 3.255, 95% CI (2.209–4.794)], consistent with the findings of Wang (11). As a vital physiological barrier, compromised oral mucosal integrity increases susceptibility to pressure injuries induced by intubation and related devices. Intubated patients often present with oral dryness, bacterial colonization, and excessive secretions (28). Research has shown that integrating a modified Beck oral assessment into oral care for ventilated patients not only reduces the incidence of ventilator-associated pneumonia but also enhances oral hygiene and reduces mucosal complications (29). Despite consensus on the importance of oral care, maintaining mucosal cleanliness in intubated patients remains challenging due to concerns about tube dislodgement and insufficient translation of nursing knowledge into practice. Therefore, standardizing oral care protocols and increasing nursing staff training are urgently needed. Moreover, intubated patients often experience thirst and dry mouth. Recent studies have demonstrated that cold water sprays can significantly alleviate these symptoms without increasing aspiration risk (30). In summary, ICU nurses should adopt a multifaceted approach—including meticulous oral care, the use of cold saline sprays, and prompt removal of secretions—to improve mucosal health and patient comfort.

#### 4.2.5 Platelet count

This study identified low platelet count as an independent risk factor for OMPI in intubated patients, with patients having platelet levels < 50 × 10^9^/L at higher risk. Although no direct causal relationship between thrombocytopenia and OMPI has been established, impaired hemostasis and the additive effect of mechanical trauma are likely contributors. Chinese Nursing Research (31) reported that coagulation dysfunction may delay tissue repair and exacerbate mucosal damage. Platelets are central to hemostasis and endothelial repair through adhesion and aggregation cascades. In thrombocytopenic patients, increased capillary fragility predisposes the oral mucosa to persistent bleeding even after minor trauma. Continuous shear forces from intubation and routine oral care may act synergistically, leading to recurrent blood blisters and scabs, particularly in the lips and oral cavity. These lesions may be painful and difficult to heal. Low platelet levels impair microvascular integrity and mucosal repair capacity. Insufficient platelet-mediated endothelial protection increases the likelihood of ischemia and microbleeding under continuous tube pressure, thereby accelerating mucosal breakdown in thrombocytopenic patients. Therefore, oral care for such patients should be performed gently, with possible use of mucosal protectants or hydrocolloid dressings to promote healing and reduce further injury (32).

### 4.3 Scientific validity and clinical value of the prediction model

In this study, a risk prediction model for OMPI in patients undergoing orotracheal intubation was developed based on multivariate logistic regression analysis incorporating five independent predictors. The model was internally validated using 1,000 bootstrap resampling iterations. The receiver operating characteristic (ROC) curve and calibration plot demonstrated favorable discrimination and calibration. External validation yielded an area under the ROC curve (AUC) of 0.854, with an overall prediction accuracy of 83.7%, suggesting good generalizability. DCA showed a net clinical benefit within the threshold probability range of 0.03–0.10, indicating high clinical utility of the nomogram. Similar conclusions were drawn in recent international research, where device-related pressure injury models demonstrated comparable discriminative performance and emphasized early risk identification in ICU patients (33, 34). This model enables clinicians to identify high-risk patients early and implement targeted interventions, such as optimizing tube fixation and sedation management, to reduce OMPI incidence in ICU settings. Recent studies (4, 5, 28) have emphasized that integrating predictive models into ICU nursing workflows can significantly lower the incidence of device-related mucosal injuries by improving early recognition and personalized care. In clinical practice, a predicted probability above 0.48, identified as the optimal cutoff by ROC analysis, indicates a high risk of OMPI. At this level, clinicians should enhance preventive care by adjusting tube fixation, using protective materials, and increasing the frequency of oral mucosal assessment to reduce pressure and friction injuries. Timely risk stratification guided by this model may reduce the incidence of OMPI and improve the overall quality of ICU nursing care. Future integration into electronic nursing systems could further enhance real-time risk monitoring and preventive decision-making. Similar risk factors have also been reported in device-related injuries caused by nasogastric tubes, oxygen masks, and urinary catheters, indicating that OMPI shares common pathophysiological mechanisms with other mucosal pressure injuries and reinforcing the generalizability of this model.

### 4.4 Limitations

This study has several limitations. First, both the modeling and validation cohorts were derived from the same institution, and the moderate sample size may limit generalizability. In addition, potential unmeasured confounders such as oral care frequency and operator variability might have influenced the results. Future research should adopt multicenter, large-scale study designs to enhance the external validity and applicability of the model. Second, the use of convenience sampling may introduce selection bias, affecting the representativeness of the study population. Third, although the model was constructed using logistic regression, it did not account for certain potential influencing factors, such as psychological status, family support, and other social determinants, which may also contribute to the development of OMPI. Finally, despite internal and external validation efforts, the long-term clinical effectiveness of the model remains untested. Prospective studies are needed to further verify and optimize the model’s performance in real-world clinical settings.

## 5 Conclusion

Based on clinical data from ICU patients undergoing tracheal intubation, this study developed a prediction model for OMPI. The model incorporates five independent risk factors—duration of intubation, use of mouthguards, RASS score, BOAS score, and platelet count—and was constructed through logistic regression and visualized using a nomogram. Both internal and external validations demonstrated strong predictive performance, with AUCs of 0.888 and 0.854, respectively, and high sensitivity and specificity, indicating excellent discriminatory power and calibration. The DCA further supported the model’s clinical value. Despite these strengths, the study has limitations, including its single-center origin and the omission of certain potential risk factors. Future research should focus on validating the model in multicenter settings with larger sample sizes and enhancing its applicability through refinement and broader clinical integration.

## Data Availability

The original contributions presented in this study are included in this article/supplementary material, further inquiries can be directed to the corresponding author.
